# Fast decomposed method to devise broadband polarization-conversion metasurface

**DOI:** 10.1038/s41598-023-35260-y

**Published:** 2023-05-20

**Authors:** Xiaofei Xiao, Jinyou Lu, Fatima Alzaabi, Mahra Almheiri, Vincenzo Giannini, Tadzio Levato

**Affiliations:** 1 Technology Innovation Institute, P.O. Box 9639, Building B04C, Masdar City, Abu Dhabi United Arab Emirates; 2 Instituto de Estructura de la Materia (IEM-CSIC), Consejo Superior de Investigaciones Científicas, Serrano 121, 28006 Madrid, Spain; 3 Centre of Excellence ENSEMBLE3 sp. z o.o., Wolczynska 133, 01-919 Warsaw, Poland

**Keywords:** Metamaterials, Metamaterials

## Abstract

Designing a broadband, wide-angle, and high-efficient polarization converter with a simple geometry remains challenging. This work proposes a simple and computationally inexpensive method for devising broadband polarization conversion metasurfaces. We focus on a cross-shape configuration consisting of two bars of different lengths connected at the center. To design the metasurface, we decompose the system into two parts with two orthogonally polarized responses and calculate the response of each part separately. By selecting the parameters with a proper phase difference in the response between the two parts, we can determine the dimensions of the system. For designing broadband polarization conversion metasurfaces, we define a fitness function to optimize the bandwidth of the linear polarization conversion. Numerical results demonstrate that the proposed method can be used to design a metasurface that achieves a relative bandwidth of $97\%$ for converting linearly polarized waves into cross-polarized waves. Additionally, the average polarization conversion ratio of the designed metasurface is greater than $91\%$ over the frequency range of 10.9–28.5 GHz. This method significantly reduces the computational expense compared to the traditional method and can be easily extended to other complex structures and configurations.

## Introduction

The polarization state of an electromagnetic wave is a fundamental characteristic that describes the direction of oscillation of its electric component. Due to the valuable information about the environment imaged (usually unavailable in other characteristics), polarization detection plays a vital role in many applications ranging from medicine, biology, and astronomy^1–5^. By contrast, manipulating the polarization of the electromagnetic wave is crucial due to the inherent sensitivity of numerous fascinating phenomena to the polarization^6–9^. However, conventional devices using bulky materials hinder miniaturization and limit their application in integrated systems.

Metamaterials are artificial materials composed of sub-wavelength structures that can produce unprecedented electromagnetic properties derived from the structures rather than the composite materials^10–21^. Metasurfaces, as a representative sample of metamaterials, consist of subwavelength structures on two-dimensional surfaces, providing an effective strategy to manipulate electromagnetic waves, including polarization and profile, with numerous advantages, including low loss and cost^22–32^. Polarization converters have been achieved using the birefringence effect of anisotropic metamaterials and the optical activity of chiral metamaterials^32–37^. Substantial efforts have been devoted to expanding the working bandwidth and enhancing conversion efficiency. Various structures, including cut-wire, ring or dick cavities, double V-shaped patches, and more complex structures, have been explored^37–42^. Polarization-conversion metasurfaces have been used in varies applications^37,43,44^, such as radar systems, satellite communication, and system requiring THz and GHz wireless communication. However, achieving a polarization converter with the ultra-broadband, wide-angle, high-efficiency, and simple geometry simultaneously remains challenging^40,45,46^.

To develop a broadband polarization converter using the traditional method, various parameters, such as patch side lengths, arc angles, and bar lengths, are swept to optimize the performance of a system across a target frequency range^44,46–49^. However, this approach is computationally expensive and time-consuming. Therefore, there is a need for a simpler method to design a broadband polarization converter. Additionally, recent publications have highlighted the misinterpretation of linear reflective polarization converters as absorbers due to the cross-polarization reflection coefficient^50,51^. A detailed theory for polarization converters can help avoid such misinterpretation in simulations and experiments.

The incident wave can be divided into two orthogonally polarized counterparts for anisotropic metasurfaces. However, traditional simulations treat the two counterparts as a single system, requiring the simultaneous tuning of multiple system parameters to simulate polarization conversion. In our method, we decompose the system into two parts and calculate each counterpart separately, significantly reducing computation cost. Once we obtain the separate responses, we determine the dimensions of the system by selecting parameters with a proper phase difference between the two parts. We define a fitness function to predict the bandwidth of the linear polarization conversion, allowing us to devise ultra-broadband polarization-conversion metasurfaces. Numerical results demonstrate that our method can guide the development of a polarization-conversion metasurface in the frequency range of 10.1–29.1 GHz with a relative bandwidth of $97\%$. The average polarization conversion ratio for the frequencies between 10.9 and 28.5 GHz is over $91\%$. The proposed metasurface is promising for many applications, such as radar systems, satellite communication, and system requiring THz and GHz wireless communication. The proposed method is instructive and straightforward and can also be extended to other complex structures, such as H-shaped metasurfaces, for which we can decompose the nearfield coupling. While our work focuses only on the reflection configuration and linear polarization conversion, the theory and method for the transmission configuration and other kinds of polarization conversion can be derived similarly with little difficulty.

## Linear optical response of the system

**Figure 1:**
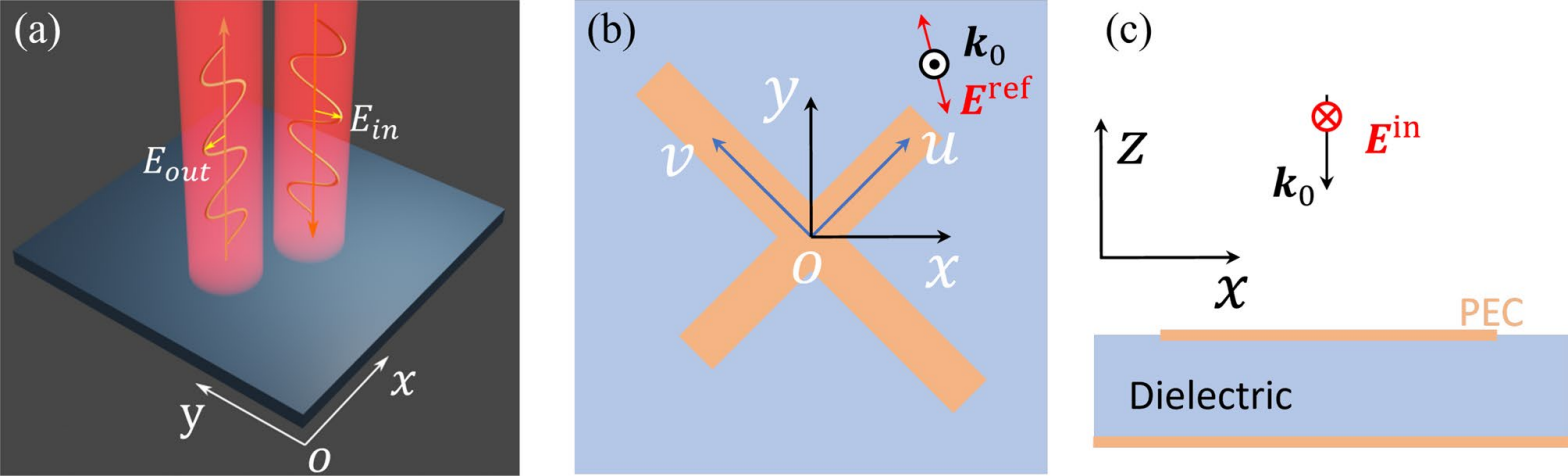
Schematic of a linear polarization converter. (**a**) Schematic of linear polarization metasurface. (**b**) The unit cell of a simple cross-shape configuration consists of two bars of different lengths connected at the center at an angle of 45$^\circ$. (**c**) The side view of the linear polarization converter. A metallic layer backs the dielectric substrate. Without loss of generality, we use a perfect electric conductor (PEC) boundary representing the metallic material. In this paper, we consider the incidence is from the top of the linear polarization converter, with the electric field along the *y*- direction unless specially stated. All calculations are performed using periodic boundary conditions in the *x* and *y* directions. Perfectly matched layer boundary conditions and perfect electric metal boundary conditions are applied in the positive and negative *z* directions, respectively.

Matrix operators can be used to analyze the linear optical response of the system (see Supplementary Material). As illustrated in Fig. 1a–c, we can derive the reflected wave using matrix multiplication (when the incidence is assumed to be linearly polarized in *y*- direction along the negative *z*- direction), as follows

1 $$\begin{aligned} {\textbf {E}}^{\textrm{ref}}=\left[ \begin{matrix}{\widetilde{R}}_{xy}\\ {\widetilde{R}}_{yy}\\ \end{matrix}\right] = \frac{E_0}{2}\left[ \begin{matrix}R_{uu}e^{i\Phi _u\ }-R_{vv}e^{i\Phi _v\ }\\ R_{uu}e^{i\Phi _u\ }+R_{vv}e^{i\Phi _v\ }\\ \end{matrix}\right] , \end{aligned}$$

where ${\widetilde{R}}_{xy}$ and ${\widetilde{R}}_{yy}$ are the cross- and co-polarization reflection coefficients, respectively, $\Phi _u$ and $\Phi _v$ are the polarized reflection phases for the *u*- and *v*-polarized normal incidence, respectively, $R_{uu}$ and $R_{vv}$ are the reflection amplitudes for the *u*- and *v*-polarized normal incidence, respectively, and $E_{0}$ is the incidence amplitude of the *y*-linear polarization. The linear polarization conversion efficiency can be described using the polarization conversion ratio (PCR) as follows

2 $$\begin{aligned} PCR=\frac{|{\widetilde{R}}_{xy}|^2}{|{\widetilde{R}}_{xy}|^2+|{\widetilde{R}}_{yy}|^2}.\ \end{aligned}$$

To simplify the analysis that follows, we assume that the system is lossless and that there is no transmission, which implies $R_{uu}=R_{vv}=1$ and $|{\widetilde{R}}_{xy}|^2+|{\widetilde{R}}_{yy}|^2=1$. As a result, PCR can be determined by varying the phase difference between $\Phi _u$ and $\Phi _v$.

Based on the aforementioned discussion, it can be concluded that a system that is symmetrical with respect to the incident polarization direction would exhibit either absorption (a large amount of loss) or reflection (a small amount of loss), as there is no cross-polarization reflection. When the system’s symmetry breaks, the reflection will contain co- and cross-polarization components. Depending on the loss and polarization conversion efficiency, the system can behave as a polarization converter, absorber, or reflector. However, cross-polarization conversion is ignored in several symmetry-breaking structures, leading to the misinterpretation of linear reflective polarization converters as absorbers^50,51^.

Under the orthogonally polarized incidence (the *u*- and *v*-polarized normal incidence), the excited modes occur on different parts of the system and are orthogonal to each other. Therefore, we can decompose the system into two parts and calculate the response of each counterpart separately, significantly reducing computation expense. After obtaining the separate responses, we can determine the dimensions of the system by selecting the parameters with an appropriate phase difference between the two parts. In this work, we use bar antennas, which readily satisfy the above assumption and result in a much lower computational burden than the conventional sweeping method.

In practical applications, it is often necessary to achieve both broadband and high-efficiency performance. Since the response of a bar antenna is smooth, we can reduce computation costs by considering the response at only a few frequencies and a small number of geometry parameters, and then fitting the entire response spectrum. By decorating or deforming structures, it is possible to achieve broadband and high-efficiency performance with multiple resonance phenomena. It is important to note that the method described here can also be applied to the transmission case, taking into account the different amplitudes in the orthogonal directions.

## Fast decomposed method to devise broadband polarization-conversion metasurface

This paper aims to provide a simple and computationally inexpensive method for devising metasurfaces with broadband polarization conversion. In the following, we will show this method step by step.

### Phase and amplitude of scattered light from a bar antenna

The phase and amplitude of the wave scattered from an antenna can significantly change across a resonance. One can easily tune the phase shift and amplitude by spatially tailoring the antenna’s geometry. In practice, the choice of the antenna can range from regular cuboids to complex three-dimensional structures. In this paper, we focus on the former due to their widely tailorable properties, design intuition, and ease of fabrication.

**Figure 2:**
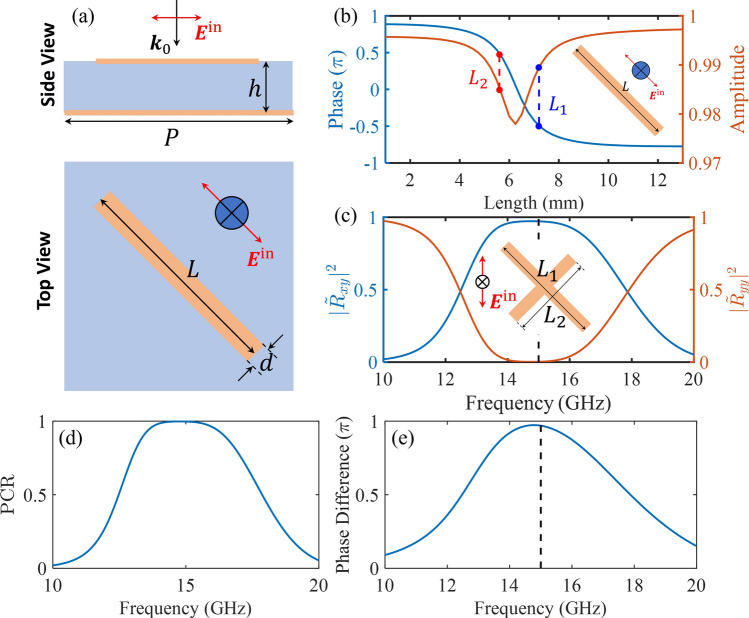
Unit cell design of a linear polarization converter for a single wavelength. (**a**) Schematic of single PEC bar antenna. (**b**) Simulated phase and amplitude of the reflected wave against the bar length for the configuration shown in (**a**). The incidence is polarized along the bar axis. (**c**) The co- and cross-polarization reflectivity ($|{\tilde{R}}_{yy}|^2$ and $|{\tilde{R}}_{xy}|^2$) under the *y*-polarised incidence. The inset shows the geometry parameters and the incidence. The corresponding response of bars with lengths of $L_1$ and $L_2$ is indicated in (**a**). (**d**) Simulated polarization conversion ratio. (**e**) The phase difference between $\Phi _{u}$ and $\Phi _{v}$ is calculated under *u*- and *v*-polarization, respectively. In these simulations, the following parameters are used: $P = {10}\,\text {mm}$, $h = {2.5}\,\text {mm}$, $d = {0.4}\,\text {mm}$, $L_{1} = {7.2}\,\text {mm}$, and $L_{2} = {5.6}\,\text {mm}$. The vertical dashed lines represent the target frequency at 15 GHz. The dielectric layer considered in this work is F4B-2 with relative permittivity of 2.65 and a loss tangent of 0.003. The original parameters (*h*, *d*, and the relative permittivity of F4B-2) used in this work have been shown to match the experimental data very well in a previous publication^44^.

Fig. S1b shows the phase and amplitude of the wave scattered from a straight rod antenna, which was found to have a phase that changes within a range of $\pi$, making it incapable of achieving $100\%$ polarization conversion. One solution to this issue is to leverage the advantages of a V-shape antenna^52^. However, using wires with different lengths in different directions can cause a significant difference in amplitude. As an alternative solution, we combine straight wires with a perfect electric metal (PEC) mirror to extend the phase difference beyond $\pi$ and achieve high reflection amplitude (the loss is due to the absorption of the dielectric layer), as shown in Fig. 2. We study the linear optical response of the structures under illumination with the polarization along their symmetrical axis, as shown in Fig. 2a. The calculated phase and amplitude of the reflected wave are shown in Fig. 2b.

### Unit cell design at a single wavelength

We focus on a simple case with a cross-shape configuration consisting of two bars of different lengths connected at the center at an angle of 45$^\circ$, as shown in Fig. 1b. Since the reflection amplitude is close to $100\%$, to achieve high polarization conversion, one only needs to ensure that the phase difference between the two bars (as shown in Fig. 2c) is $\pi$. Based on this criterion, the lengths of the two bars are selected, $L_{1} = {7.2}\,\text {mm}$ and $L_{2} = {5.6}\,\text {mm}$, for a target frequency of 15 GHz.

As shown in Fig. 2c,d, the co- and cross-polarization reflectivity and the PCR under the *y*-polarized incidence indicate that we realized a high linear polarization conversion around the target frequency based on the proposed method. To verify the phase difference between $\Phi _{u}$ and $\Phi _{v}$, we calculated this value by simulating the system’s response under *u*- and *v*-polarization, respectively. Fig. 2e confirms the validation of our method. The small deviation is because we truncated the lengths to $L_{1} = {7.2}\,\text {mm}$ and $L_{2} = {5.6}\,\text {mm}$ to approximately achieve the $\pi$ phase difference, and the changes of the configuration because of the adding of the perpendicular bar compared to the single bar case in Fig. 2a.

**Figure 3:**
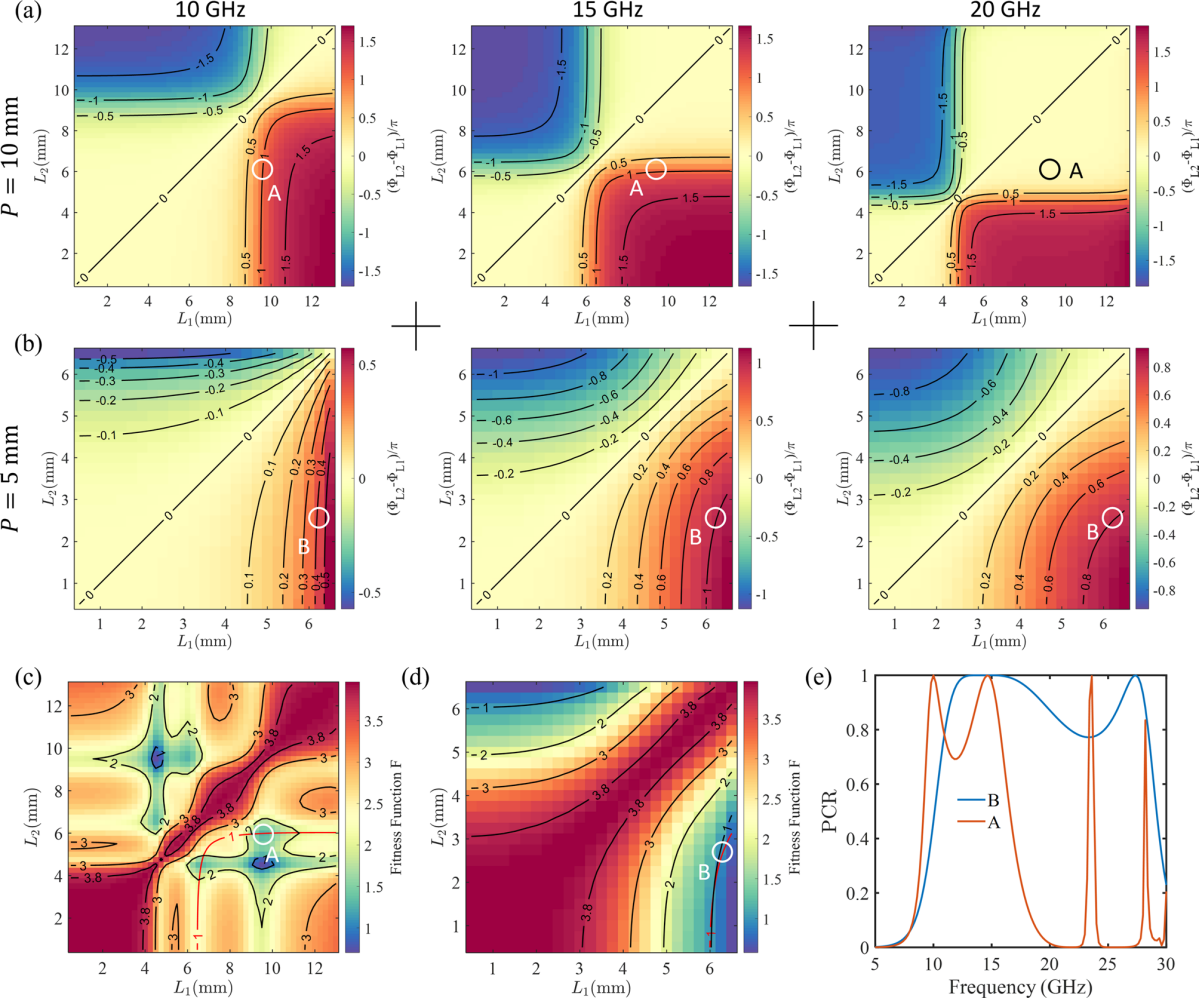
Design of linear polarization converters at multiple wavelengths. (**a**) and (**b**) Maps and contours of the phase difference of $(\Phi _{L_{2}}-\Phi _{L_{1}})/\pi$ against the bar lengths, where $\Phi _{L_{i}}$ is the absolute phase calculated using the same method shown in Fig. 2b. The configuration is the same as Fig. 2a except the period (a, $P = {10}\,\text {mm}$; b, $P = {5}\,\text {mm}$) and the frequency of interest (at 10 GHz, 15 GHz, and 20 GHz). (**c**) and (**d**) Maps of the fitness function *F* calculated using Eq. 3 for two periods (c, $P = {10}\,\text {mm}$; d, $P = {5}\,\text {mm}$). The red contour lines for the phase difference of $\pi$ at the frequency of 15 GHz are also marked. (**e**) The PCR under the *y*-polarised incidence for cases A and B in c and d. The configuration is the same as Fig. 2c except for the dimensions. In these simulations, the following parameters are used. Case A: $P = {10}\,\text {mm}$, $L_{1} = {9.5}\,\text {mm}$, and $L_{2} = {6}\,\text {mm}$. Case B: $P = {5}\,\text {mm}$, $L_{1} = {6.25}\,\text {mm}$, and $L_{2} = {2.5}\,\text {mm}$. In all cases, the values of *h* and *d* are fixed at ${2.5}\,\text {mm}$ and ${0.4}\,\text {mm}$, respectively.

Once the phase difference and amplitude requirements are guaranteed, the choice of the two lengths can be various. To show all possible choices, we transform the curve in Fig. 2b into a two-dimensional map in Fig. 3a (at 15 GHz). These maps correspond to the phase difference of $(\Phi _{L_{2}}-\Phi _{L_{1}})/\pi$, where $\Phi _{L_{i}}$ is the absolute phase shown in Fig. 2b. Therefore, any choice close to the contour line at 1 would be an alternative solution.

### Unit cell design at multiple wavelengths

The former configuration realizes linear polarization converters for a narrow-band range. However, we did not consider the complex structure and other parameters, such as the thickness of the dielectric layer and period, which offer us more tunability of the performance bandwidth. In practice, there are various ways to widen the performance bandwidth. This part introduces an intuitive and straightforward way to achieve this.

The key to achieving a broadband linear polarization converter is to maintain a $\pi$ phase difference between the two orthogonal polarizations over a broadband frequency range. In our case, the reflection amplitude is close to 1 (reflection mode), allowing us to ignore the mismatch of the reflection amplitude for cross-polarization^52^. This significantly reduces the design difficulty.

Figure 3a,b show the phase difference of $(\Phi _{L_{2}}-\Phi _{L_{1}})/\pi$ at different frequencies with different periods. As discussed above, one can readily design a linear polarization converter at a target frequency by selecting a solution close to the contour line at 1. The smaller gradient around the solution corresponds to a broader bandwidth performance because it indicates a flatter spectrum around the target wavelength.

Here, we propose a method to achieve a broadband polarization converter. Figure 3a,b suggest that the performance can be tuned by varying other parameters, such as the period in our case. To predict the bandwidth more precisely, we define a fitness function that reads

3 $$\begin{aligned} F = \frac{1}{N}\sum _{n=1}^{N} |R_{L_{2}}^{n} \exp (-i\Phi _{L_{2}}^{n})+R_{L_{1}}^{n} \exp (-i\Phi _{L_{1}}^{n})|^2 , \end{aligned}$$

where $n=1,2,3$ ($N=3$) corresponds to frequencies of 10 GHz, 15 GHz, and 20 GHz, respectively, and $R_{L_{j}}^{n}$ and $\Phi _{L_{j}}^{n}$ ($j=1,2$) denote the refection amplitude and phase at different frequencies. Figure 3c,d show the calculated fitness function for two periods. A smaller fitness function value and smaller gradient correspond to a broader spectrum. We expect the PCR to be close to 1 at the target frequency (15 GHz). To achieve this, we could assign a larger weight to the phase difference at the target frequency when calculating the fitness function. However, we take an alternative strategy here: We overlap the contours of the phase difference at 15 GHz and the fitness function. Then, we choose the dimension parameters that allow the value of the fitness function to be close to both the minimum value of the fitness function and the $\pi$ phase difference at 15 GHz.

Using the method described above, we select two cases labeled as A and B in Fig. 3c,d. Fig. 3e shows the performance for the two cases, verifying the proposed method. Both configurations exhibit high linear polarization conversion at the target wavelength (15 GHz). Configuration B shows a much broader bandwidth performance than configuration A because the fitness function value of configuration B is smaller, and the gradient of the fitness function around this choice is minor. Results in Fig. 3e also indicate that parameters such as the period offer us more tunability in the performance bandwidth. Thus, the map (the information including the phase difference and the gradient) in Fig. 3a and b can be used to predict the performance. For example, from Fig. 3b, we can conclude that the polarization conversion at 10 GHz is not high compared to the case at 15 GHz since, for 10 GHz, the phase difference is only around 0.4$\pi$ and the gradient is not tiny.

**Figure 4:**
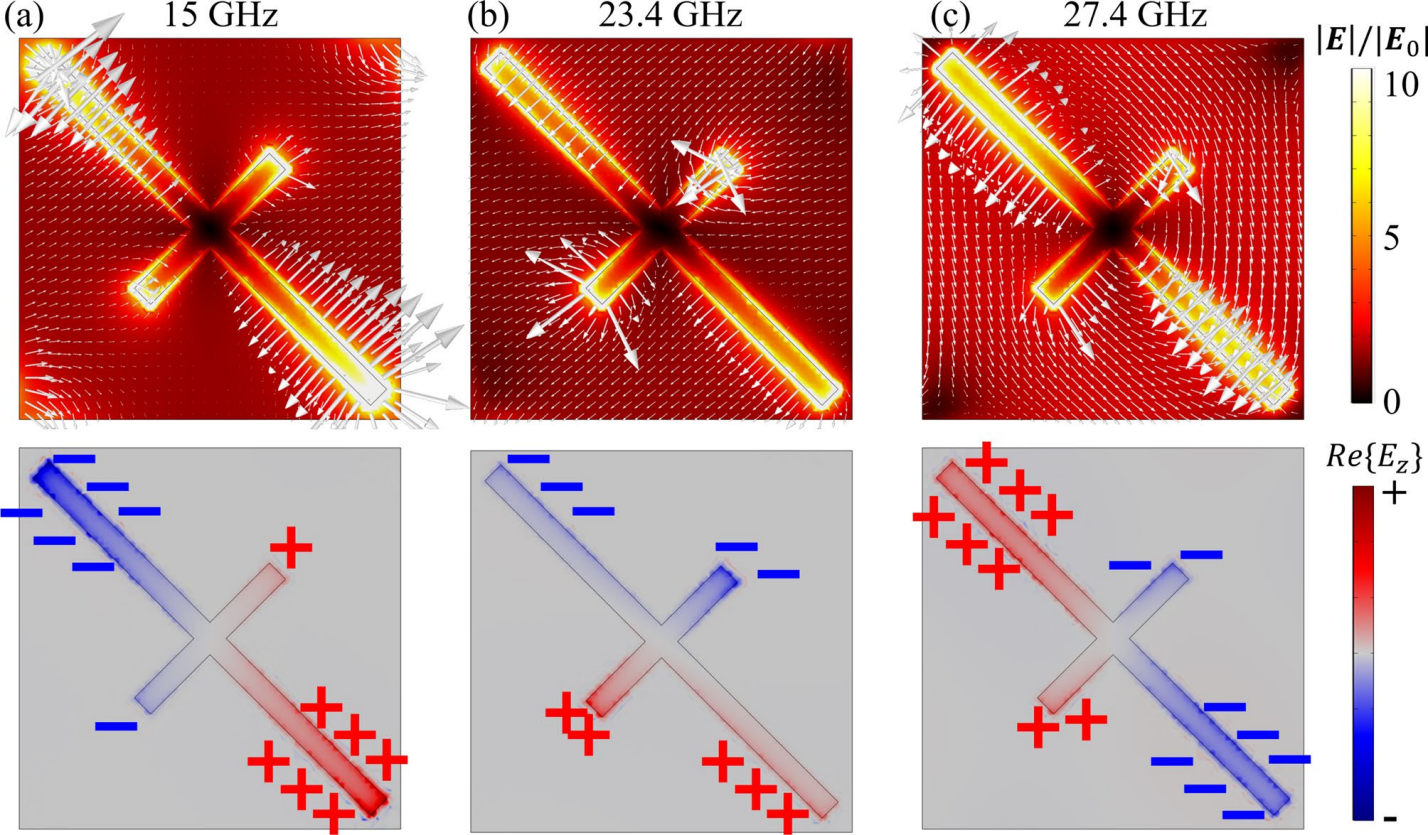
Near-field plots for three different frequencies. Distribution of the electric field magnitude and electric near field (arrow direction denotes the direction of the vector of [Re{E_x_}, Re{E_y_}, Re{E_z_}], and its length denotes the amplitude of the near field) in the top panel and the real part of the *z*-component of the near field ($E_z$) in the bottom panel on the metallic parts of the metasurface unit cell at frequencies of (**a**) 15 GHz, (**b**) 23.4 GHz and (**c**) 27.4 GHz. The electric field is normally incident along the *y*-direction. In the following simulations, $P = {5}\,\text {mm}$, $L_{1} = {6.25}\,\text {mm}$, $L_{2} = {2.5}\,\text {mm}$, $h = {2.5}\,\text {mm}$, and $d = {0.4}\,\text {mm}$.

The near field distributions for configuration B are shown in Fig. 4 for three frequencies (15 GHz, 23.4 GHz, and 27.4 GHz), corresponding to the peaks and the dip in the PCR plot. For the frequencies to the peaks (15 GHz and 27.4 GHz), the plots illustrate that the “hot spots” are primarily distributed around the ends of the rods, indicating the resonance of the metallic bars under the corresponding illumination conditions. The *z*-component of the near field ($E_z$) in Fig. 4a,c confirms that the two orthogonal modes have a $\pi$ phase difference (i.e., they are out-of-phase). The enhancement for the 23.4 GHz frequency is comparatively lower than the enhancement for the other two cases. In this instance, the *z*-component of the near-field indicates that the $\pi$ phase difference requirement does not hold well between the two orthogonal modes, leading to a relatively low polarization conversion, as expected.

In this way, we can achieve a broadband polarization conversion metasurface by using the selected parameters in Fig. 3d. The average PCR is approximately $91\%$ over the frequency range of 10.9–28.5 GHz, with a local minimum value at 23.4 GHz. The full width at half-maximum is 19 GHz (10.1–29.1 GHz). It should be noted that the performance can be further improved by considering complex structures and tuning other parameters.

### Performance for oblique incidence

**Figure 5:**
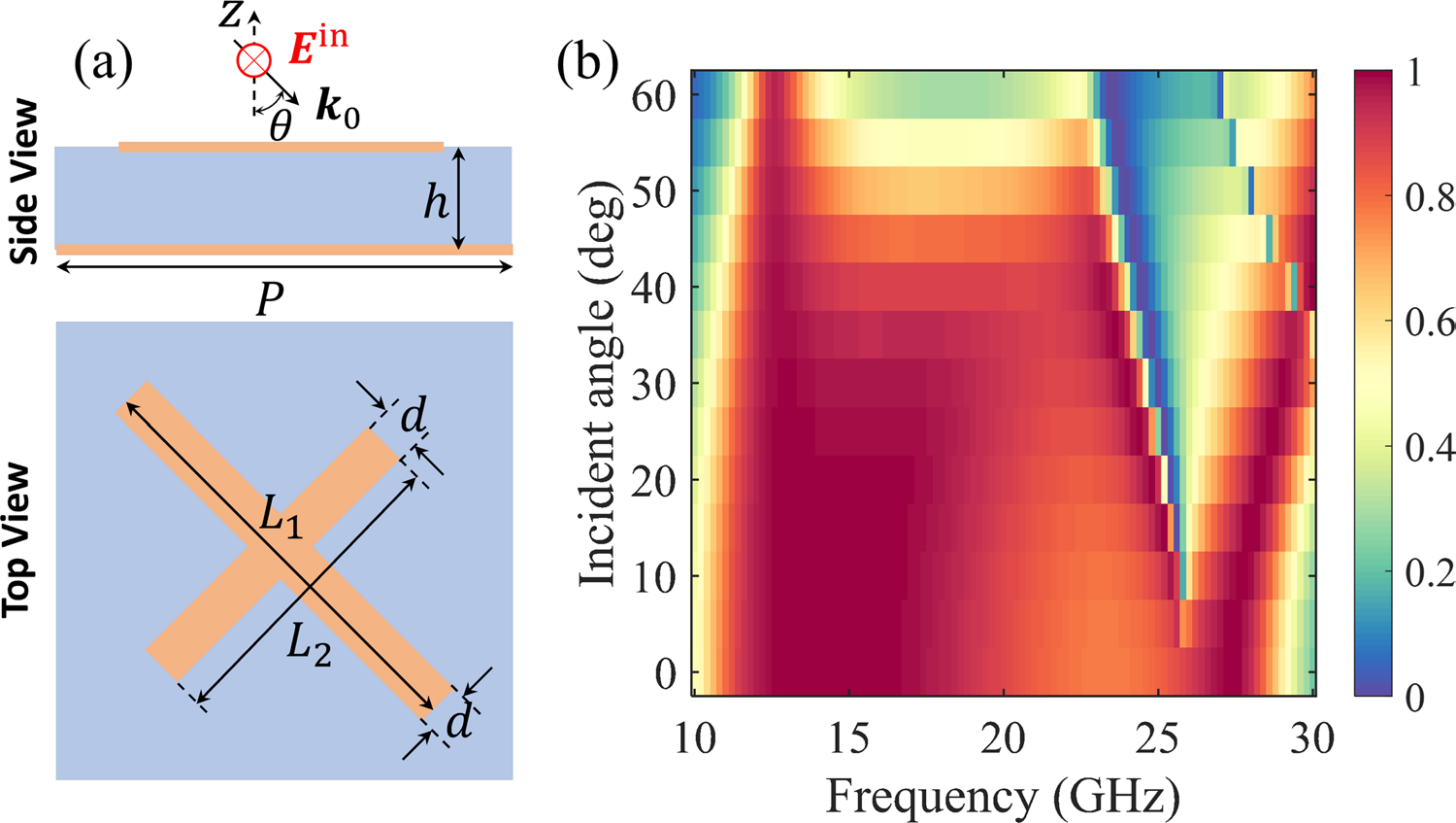
Polarization-conversion performance for different incident angles. (**a**) Schematic of the configuration. $\theta$ denotes the incident angle. The incident electric field is in the *y*-direction all the time. In the following simulations, $P = {5}\,\text {mm}$, $L_{1} = {6.25}\,\text {mm}$, $L_{2} = {2.5}\,\text {mm}$, $h = {2.5}\,\text {mm}$, and $d = {0.4}\,\text {mm}$. (**b**) Simulated PCR map as a function of the frequency and incident angle.

It is essential to evaluate the performance of the polarization converter under angled incidence. Figure 5 shows the configuration and the simulated PCR map as a function of the frequency and the incident angle. The spectra become dual-band as the angle increases. For the first band, the PCR between 10.5 and 24.5 GHz exhibits only minor changes with an increasing incident angle when the angle is smaller than 50$^\circ$; however, at higher frequencies, the PCR decreases drastically due to the grating diffraction effect. As the incident angle increases, the central frequency of the second band (around 28 GHz) shifts to higher frequencies while maintaining a bandwidth of around 3.5 GHz. Thus, the proposed polarization converter performs well for large oblique incidence angles in the range of 10.5–24.5 GHz and can be easily tuned using the proposed method.

It is worth noting that our theoretical model neglects the coupling effect between the two orthogonal directions (u- and v-axis). This is a reasonable assumption for normal incidence and oblique incidence with small incident angles. However, as the incident angle increases, this approximation becomes less accurate, which results in a degradation in the performance for oblique incidence.

## Discussions

The proposed method takes great computation advantages over the traditional method. It can significantly reduce the computation expense by decomposing the system into two parts and calculating the two counterparts separately. In the traditional method, we would need to calculate $N_{1} \times N_{2} \times N_{{\text{f}}}$ cases to obtain the optimal response of the system, where $N_1$ and $N_2$ are the numbers of the swept values of each parameter, and $N_{{\text{f}}}$ is the number of frequency points calculated. However, in the proposed method, we only need to calculate $(N_{1} + N_{2} ) \times N_{{\text{f}}}$ times, and $N_{{\text{f}}}$ could be a much smaller integer than in the traditional method (in this paper, $N_{{\text{f}}} = 3$). The computation expense can be further reduced by considering the smooth shape of the response in Fig. 2b since we can extrapolate the data to get the full spectrum, which is very challenging for the traditional method. If the constituent parts in the two orthogonal directions are the same, the number of the simulations is reduced to only $3N_1$ (such as the configuration shown in Fig. 3).

For example, in the traditional method, we would need to calculate $N_{1} \times N_{2} \times N_{{\text{f}}} = 60 \times 60 \times 100 = 360000$ times (assuming there are 60 swept values of the lengths and 100 frequency points for a range of 10–30 GHz). In our method, we only need to calculate $3N_1=3\times 10=30$ times (considering the data extrapolation). The computational speed of our method is 12000 times that of the traditional method.

Furthermore, in Table 1, we compared the processing time required for designing a broadband polarization-conversion metasurface (Case A in Fig. 3e) using the conventional and proposed methods. The results show that the proposed method is approximately 20000 times faster than the traditional method. This is because cross-structure simulations require a larger mesh volume to resolve the structure than bar antenna simulations.

**Table 1:** Processing time for designing broadband polarization-conversion metasurface (Case A in Fig. 3e) with the conventional and proposed methods.

Method	$N_1$	$N_2$	$N_{{\text{f}}}$	Time (Hour)
Traditional	60	60	100	9805.5
Proposed	10	–	3	0.5

The simulations were performed using COMSOL 5.6 on a workstation with a 3.20 GHz Intel Xeon Silver 4215R CPU and 128 Gb RAM. Finer mesh was used in our simulation. $N_1$ and $N_2$ represent the numbers of swept values of $L_1$ and $L_2$, respectively, while $N_{{\text{f}}}$ represents the number of calculated frequencies. (Note: for the traditional method, we simulated only with one pair of $L_1$ and $L_2$ to estimate the running time for each pair of $L_1$ and $L_2$. The total running time was then obtained by multiplying that time by $N_1$ $\times$ $N_2$).

Although the system cannot be decomposed, we can divide its response into two orthogonally polarized counterparts for a few frequencies and use the fitness function to predict the bandwidth. This work focuses on the reflection configuration and linear polarization conversion. However, the theory and method for the transmission configuration and other types of polarization conversion can be derived in a similar manner.

The performance of the polarization converter can be further improved by optimizing the parameters, including the thickness and the period. An alternative way to expand the bandwidth is to combine structures^33^ resonant at different wavelengths (and carefully tune the coupling when combining them) and to explore multiple order modes of the system by tailoring the geometry of the structures^44^. The polarization purity can be enhanced by adding gratings above the structures^43,44^, which could be used to select the polarization of the incident and reflected waves.

Compared to other polarization converters, the proposed one performs better with simple geometry. Table S1 compares the proposed converter and other linear polarization converters. It can be observed that the proposed converter, with a reasonable relative thickness, has an ultra-wide operating frequency band in which the average PCR is greater than $91\%$, demonstrating better performance.

We employed full-wave simulations for Maxwell’s equations (based on the finite element method) to evaluate the performance of the designed metasurface and validate our proposed method. While the measured PCR values in experiments may differ from the full-wave simulation results, these discrepancies are primarily attributed to experimental imperfections, which is out of the scope of this paper. Further analysis of the differences could benefit the design of broadband polarization-conversion metasurfaces.

## Conclusion

This paper proposes a straightforward and informative method for devising broadband polarization conversion metasurfaces. The proposed method circumvents the costly computation involved in the traditional method by decomposing the system into two parts and separately calculating the response of each part. The dimensions of the system can be selected based on the phase difference in the response between the two parts to achieve a high PCR. A fitness function is defined to predict the bandwidth of the linear polarization conversion. We demonstrated that the proposed method can guide us to readily devise a metasurface with broadband polarization conversion while requiring significantly less computation than the traditional method.

As an example, we design a polarization-conversion metasurface operating in the frequency range from 10.1 to 29.1 GHz with a relative bandwidth of $97\%$. The average PCR for the frequencies from 10.9 to 28.5 GHz is over $91\%$, demonstrating its better performance than other polarization converters. The proposed metasurface has potential applications in radar systems, satellite communication, and system requiring THz and GHz wireless communication. Furthermore, the proposed method can be extended to other complex structures and other configurations.

## Supplementary Information

Supplementary Information.

## Data Availability

The data generated during and/or analyzed during the current study are available from the corresponding author on reasonable request.
