# Supplementary material for: Fast decomposed method to devise broadband polarization-conversion metasurface
Source: Sci Rep. 2023 May 20;13:8196. doi: 10.1038/s41598-023-35260-y (PMC10199941; doi:10.1038/s41598-023-35260-y)
Supplement: Supplementary file 1 — Supplementary Information. [file 41598_2023_35260_MOESM1_ESM.pdf]

Fast Decomposed Method to Devise Broadband Polarization-Conversion Metasurface

Xiaofei Xiao[1,*], Jinyou Lu[1], Fatima Alzaabi[1], Mahra Almheiri[1], Vincenzo Giannini[1,2,3], and Tadzio Levato[1]

[1]Technology Innovation Institute, P.O. Box 9639, Building B04C, Masdar City, Abu Dhabi, United Arab Emirates
[2]Instituto de Estructura de la Materia (IEM-CSIC), Consejo Superior de Investigaciones Científicas, Serrano 121, 28006 Madrid, Spain
[3]Centre of Excellence ENSEMBLE3 sp. z o.o., Wolczynska 133, Warsaw, 01-919, Poland
[*]Xiaofei.Xiao@tii.ae

1 Theory based on matrix operator

We apply matrix operators to analyze the linear optical response of the system. The Jones calculus is the mathematical formalism developed by R. C. Jones[1]. With this formalism, a Jones vector can represent the polarized incident light. A Jones matrix of the element can represent an optical element's linear optical effect on the incident polarization. Although Jones did not consider reflection from a surface or loss in the material in the original papers, we can use a similar mathematical formalism to analyze these cases.

As shown in Figure 1b, we will use two standard Cartesian coordinate systems in our analysis. The unit vector in the positive direction of each axis reads

$$\mathbf{x} = \begin{bmatrix} 1 \\ 0 \end{bmatrix}, \tag{1}$$

$$\mathbf{y} = \begin{bmatrix} 0 \\ 1 \end{bmatrix}, \tag{2}$$

and

$$\mathbf{u} = \frac{1}{\sqrt{2}}(\mathbf{x}+\mathbf{y}) = \frac{1}{\sqrt{2}}\begin{bmatrix} 1 \\ 1 \end{bmatrix}, \tag{3}$$

$$\mathbf{v} = \frac{1}{\sqrt{2}}(-\mathbf{x}+\mathbf{y}) = \frac{1}{\sqrt{2}}\begin{bmatrix} -1 \\ 1 \end{bmatrix}. \tag{4}$$

The rotation matrix from the xOy coordinate to the uOv coordinate is written as

$$\mathbf{T} = \begin{bmatrix} \cos\theta & \sin\theta \\ -\sin\theta & \cos\theta \end{bmatrix} = \frac{1}{\sqrt{2}}\begin{bmatrix} 1 & 1 \\ -1 & 1 \end{bmatrix}, \tag{5}$$

where $\theta = 45^\circ$ is the angle between the two coordinate systems.

In the uOv system, due to the spatial symmetry of the system, the reflection matrix can be written as

$$\mathbf{R}^{uv} = \begin{bmatrix} \widetilde{R}_{uu} & \widetilde{R}_{uv} \\ \widetilde{R}_{vu} & \widetilde{R}_{vv} \end{bmatrix} = \begin{bmatrix} R_{uu}e^{i\Phi_u} & 0 \\ 0 & R_{vv}e^{i\Phi_v} \end{bmatrix}, \tag{6}$$

corresponding to the reflection matrix in the xOy system

$$\mathbf{R}^{xy} = \begin{bmatrix} \widetilde{R}_{xx} & \widetilde{R}_{xy} \\ \widetilde{R}_{yx} & \widetilde{R}_{yy} \end{bmatrix} = \mathbf{T}^{-1}\mathbf{R}^{uv}\mathbf{T} = \frac{1}{2}\begin{bmatrix} R_{uu}e^{i\Phi_u} + R_{vv}e^{i\Phi_v} & R_{uu}e^{i\Phi_u} - R_{vv}e^{i\Phi_v} \\ R_{uu}e^{i\Phi_u} - R_{vv}e^{i\Phi_v} & R_{uu}e^{i\Phi_u} + R_{vv}e^{i\Phi_v} \end{bmatrix}, \tag{7}$$

where Φ_u and Φ_v are the polarized reflection phase for the u- and v-polarized normal incidence, respectively. R_{uu} and R_{vv} are the reflection amplitude for the u- and v-polarized normal incidence, respectively. The incidence is assumed to be linearly polarized in y- direction along the negative z- direction, which can be described as

$$\mathbf{E}^{\text{in}} = \begin{bmatrix} 0 \\ E_0 \end{bmatrix}. \tag{8}$$

To consider the effects of multiple surfaces, we can calculate the reflected wave using matrix multiplication as follows

$$\mathbf{E}^{\text{ref}} = \begin{bmatrix} \widetilde{R}_{xy} \\ \widetilde{R}_{yy} \end{bmatrix} = \mathbf{T}^{-1}\mathbf{R}^{uv}\mathbf{T}\mathbf{E}^{\text{in}} = \mathbf{R}^{xy} \begin{bmatrix} 0 \\ E_0 \end{bmatrix} = \frac{E_0}{2} \begin{bmatrix} R_{uu}e^{i\Phi_u} & -R_{vv}e^{i\Phi_v} \\ R_{uu}e^{i\Phi_u} & +R_{vv}e^{i\Phi_v} \end{bmatrix}, \tag{9}$$

where $\widetilde{R}_{xy}$ and $\widetilde{R}_{yy}$ are the cross- and co-polarization reflection coefficients, respectively, when we have y-polarized incidence. The linear polarization conversion efficiency can be described using the polarization conversion ratio (PCR) as follows

$$PCR = \frac{\left|\widetilde{R}_{xy}\right|^2}{\left|\widetilde{R}_{xy}\right|^2 + \left|\widetilde{R}_{yy}\right|^2}. \tag{10}$$

To simplify the analysis, we assume the system is lossless and there is no transmission, which means

$$R_{uu} = R_{vv} = 1, \tag{11}$$

and

$$\left|\widetilde{R}_{xy}\right|^2 + \left|\widetilde{R}_{yy}\right|^2 = 1. \tag{12}$$

Therefore, the PCR is determined by the phase difference between Φ_u and Φ_v, which can be achieved by adjusting the dimensions and arrangement of the metasurface elements.

2 Scattering from a linear rod antenna

We assume a thin linear antenna with a circular rod of radius a embedded in a homogeneous environment with refractive index $n_{\text{env}} = 1$, made of a perfect electric conductor. We calculate the current distribution based on the boundary condition by solving Hallén integral equations for linear antennas. Then, we perform the integration to obtain the z-component of the magnetic vector potential, from which we determine the z-component electrical field. Here, we summarized the framework of our method.

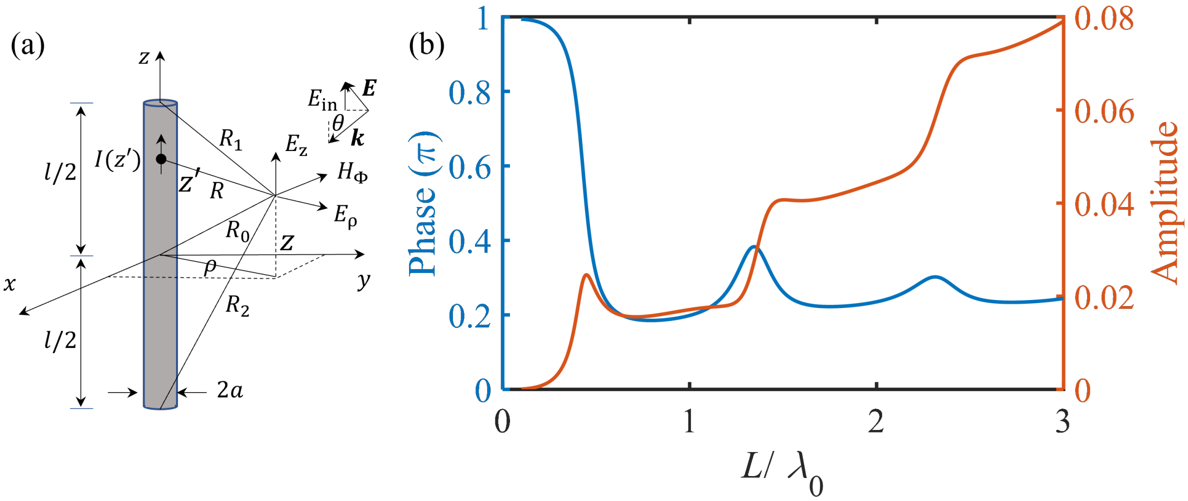

Figure S1. Scattering field calculation based on Hallén integral equation. (a) Schematic of the fields of a thin wire antenna. (b) Phase and amplitude of scattered wave from a straight wire.

The external source field E_{in} is assumed to be a uniform plane wave incident at an angle θ with respect to the z-axis on a receiving thin linear antenna. The z-component of the incident field is given by:

$$E_{\text{in}}(z) = E_0 \sin\theta e^{ikz\cos\theta}, \tag{13}$$

where E_0 is the amplitude, θ is the incident angle, k is the wavenumber. We consider the case that the incidence impinges normal to the axis of the rod and polarizes along it. That is the $\theta = \pi/2$, we get $E_{\text{in}} = E_0$.

Table S1. Comparison with other wideband polarization converters. λ_0 denotes the central wavelength of the operating wavelength range.

Reference	Structures	Operating Bandwidth	Relative Bandwidth	Operating PCR (%)	Thickness	Relative Thickness
[4]	disks and split-ring resonators	0.65 THz - 1.45 THz	76.2	≥ 80	30 μm	$\lambda_0/9.52$
[5]	dumbbell-like structure	2.026 THz - 2.481 THz	20.2	≥ 85	22 μm	$\lambda_0/6.05$
[6]	four L-shaped structure	4.2 THz - 5.2THz	21.3	≥ 98	7 μm	$\lambda_0/9.11$
[7]	cross-shaped structure	0.95 THz - 1.3 THz	34.8	≥ 99	20 μm	$\lambda_0/13.03$
[8]	double U-shaped structure	6.91 GHz - 14.31 GHz	69.7	≥ 90	3 mm	$\lambda_0/9.42$
[9]	cut-wire structure	5.1 GHz -12.1 GHz	81.4	≥ 90	4 mm	$\lambda_0/8.71$
[10]	disks and split-ring resonators	5.7 GHz - 10.3 GHz	57.5	≥ 90	2.9 mm	$\lambda_0/12.92$
**Current study**	cross-shaped structure	10.9 GHz - 28.5 GHz	89.3	≥ 91	2.5 mm	$\lambda_0/6.08$

The current distribution on a thin linear antenna can be calculated using Hallén integral equation[2]. The boundary condition on the antenna surface (i.e., at $\rho = a$) requires that the z component of the total field vanish. We obtain Hallén integral equation as follows

$$\frac{\mu}{4\pi}\int_{-l/2}^{l/2} I(z')G(z-z')\mathrm{d}z' = -i\omega\mu\varepsilon(\partial_z^2 + k^2)^{-1}E_{\mathrm{in}}(z), \tag{14}$$

where ω denotes the angular frequency of the incidence, ε and μ denote the permittivity and permeability of the surrounding, respectively, k is the wavevector, $G(z-z') = \frac{e^{-ikR}}{R}$ and $R = \sqrt{a^2 + (z-z')^2}$. Given the additional boundary condition at the ends of the antenna ($I(z = l/2) = I(z = -l/2) = 0$), we can obtain the current distribution $I(z)$ using equation 14.

Once the current distribution is calculated, the z-component of the magnetic vector potential $A_z(z,\rho)$ can be determined using the following equation[2]

$$A_z(z,\rho) = \frac{\mu}{4\pi}\int_{-l/2}^{l/2} I(z')\frac{e^{-ikR}}{R}\mathrm{d}z', \tag{15}$$

where $R = \sqrt{\rho^2 + (z-z')^2}$.

Given the Lorentz gauge, the z-component of the electric field of a thin rod antenna can be readily calculated as follows

$$E_z = \frac{\partial_z^2 A_z + k^2 A_z}{i\omega\mu\varepsilon} \tag{16}$$

We can approximate the second derivative in Equation 16 using the second-order central difference algorithm as follows:

$$f''(x_i) \approx \frac{f_{i+1} - 2f_i + f_{i-1}}{h^2} \tag{17}$$

where h is the distance between neighboring x values on the discretized domain.

We consider the vacuum case, where the relative permeability and permittivity are $\mu_r = 1$, $\varepsilon_r = 1$. At a fixed incident wavelength, the phase shift between the emitted and the incident waves of a straight-thin antenna changes dramatically across a resonance.

Figure S1b shows the phase and amplitude of the wave scattered from a straight rod antenna. However, it is concluded that the phase shift between the incident and scattered waves from a straight wire antenna changes within a range of π, which is insufficient for our purpose. One solution is to utilize the advantages of the V-shape antenna[3]. However, another issue arises in that wires with different lengths will have significantly different amplitudes. To address this, we implemented an alternative solution that combined straight wires with a PEC mirror to extend the phase difference beyond π and achieve approximately 99% reflection amplitude.

References

1. Born, M. & Wolf, E. *Principles of optics: electromagnetic theory of propagation, interference and diffraction of light* (Elsevier, 2013).
2. Orfanidis, S. J. *Electromagnetic waves and antennas* (Rutgers University New Brunswick, NJ, 2002).
3. Yu, N. *et al.* Light propagation with phase discontinuities: generalized laws of reflection and refraction. *Science* **334**, 333–337 (2011).

4. Cheng, Y. Z. *et al.* Ultrabroadband reflective polarization convertor for terahertz waves. *Appl. Phys. Lett.* **105**, 181111 (2014).

5. Meng, W. *et al.* Dynamically tunable high-efficiency broadband terahertz linear polarization converter based on dirac semimetal metamaterials. *Microw. Opt. Technol. Lett.* **62**, 2703–2707 (2020).

6. Lu, T., Qiu, P., Lian, J., Zhang, D. & Zhuang, S. Ultrathin and broadband highly efficient terahertz reflective polarization converter based on four l-shaped metamaterials. *Opt. Mater.* **95**, 109230 (2019).

7. Liu, W. *et al.* Ultra-efficiency broadband terahertz polarization converter based on a cross-shaped metamaterial. *AIP Adv.* **12**, 085101 (2022).

8. Mei, Z. L., Ma, X. M., Lu, C. & Zhao, Y. D. High-efficiency and wide-bandwidth linear polarization converter based on double u-shaped metasurface. *Aip Adv.* **7**, 125323 (2017).

9. Zhao, J. C. & Cheng, Y. Z. Ultra-broadband and high-efficiency reflective linear polarization convertor based on planar anisotropic metamaterial in microwave region. *Optik* **136**, 52–57 (2017).

10. Zhao, J. & Cheng, Y. A high-efficiency and broadband reflective 90 linear polarization rotator based on anisotropic metamaterial. *Appl. Phys. B* **122**, 1–7 (2016).
